# Gelatin and Collagen from Sheepskin

**DOI:** 10.3390/polym16111563

**Published:** 2024-05-31

**Authors:** Andrea Marie E. Matinong, Kim L. Pickering, Mark R. Waterland, Yusuf Chisti, Richard G. Haverkamp

**Affiliations:** 1 School of Food and Natural Sciences, Massey University, Palmerston North 4442, New Zealand; a.matinong@massey.ac.nz (A.M.E.M.); m.waterland@massey.ac.nz (M.R.W.); 2 School of Engineering, University of Waikato, Hamilton 3240, New Zealand; klp@waikato.ac.nz; 3 Institute of Tropical Aquaculture and Fisheries, Universiti Malaysia Terengganu, Kuala Nerus 21030, Malaysia; yusuf.chisti@umt.edu.my

**Keywords:** gelatin, collagen, extraction, sheepskin

## Abstract

**Simple Summary:**

Simple Summary: With the low demand for leather from sheepskin, New Zealand slaughterhouses dispose of most byproduct sheepskin from meat production as solid waste. This study aimed to develop a use for these sheepskins as a source of gelatin or collagen. Three extraction methods were evaluated. The extracted material was characterized for gelatin and collagen composition and structure. Sheepskin was found to be a useful alternative source of gelatin–collagen material.

**Abstract:**

Abattoirs dispose of sheepskins as solid waste due to low price and poor demand for sheepskin leather. In principle, as an alternative to being disposed of in landfill, sheepskins can serve as a source of the protein collagen or the hydrolysis product, gelatin. In this research, sheepskins collected from abattoirs were used as a source of collagen. Three extraction methods were compared: acid extraction, acid with enzymes, and alkali extraction. The extracted material was characterized using scanning electron microscopy (SEM) and Fourier-transform infrared spectroscopy (FTIR), small angle X-ray scattering (SAXS), and sodium dodecyl sulfate polyacrylamide gel electrophoresis (SDS-PAGE). The collagen and gelatin extraction yield ranged from 3.1% to 4.8% with the product purity determined by hydroxyproline, ranging from 7.8% for the alkali process to 59% and 68% for the acid and acid-enzyme processes. SDS PAGE showed that the acid process produced fragments with molecular weights in the range 100 to >250 kDa, while acid–enzyme resulted in smaller fragments, below 30 kDa. The FTIR region of the amide I band at 1800–1550 cm^−1^, which was used as an indicator of the collagen and gelatin content, showed that the gelatin dominated in the acid extracts, and the alkaline extract contained a large portion of keratin. SAXS was found to be a sensitive method for showing the presence of intact collagen fibrils in materials from all of the extraction methods, albeit at low concentrations. Herein, sheepskin is shown to be a useful source for collagen–gelatin material of varying molecular weights.

## 1. Introduction

Collagen is the most abundant structural protein in animals [1]. Collagen can be extracted from animal tissue such as skins from animals slaughtered for meat and further processed for diverse applications. Gelatin is a hydrolysis product of collagen, and maybe readily obtained from skins, sometimes in combination with collagen, wherein the two may grade into each other with a range of molecular weights.

Collagen and gelatin are used in cosmetics [2], medical and pharmaceutical products [3], and food formulation and packaging [4]. Methods used to extract collagen from tissue [5] include hydrolysis with acids [6] and alkalis [7], solubilization with salts [8], and enzymatic hydrolysis [9]. Often, these chemical treatments are assisted with ultrasound [10] and microwaves [11].

This work compares gelatin and collagen extraction from sheepskins using acid hydrolysis, acid-enzyme hydrolysis. and alkali hydrolysis. The aim was to identify a suitable process for collagen recovery from sheepskins, in the form of gelatin or collagen, for possible commercial systems.

## 2. Materials and Methods

Whole sheepskins from unknown sheep breeds were collected from a local abattoir (ANZCO Foods, Bulls, New Zealand). The sheepskins were stored at −20 °C until they were processed for extraction. Other materials and chemicals used were acetic acid (99.7%, Thermo Fischer Scientific, Waltham, MA, USA), sodium chloride (99%, Dominion Salt, Lake Grassmere, New Zealand), EDTA-4Na (Hamilton Chemicals, Hamilton, New Zealand), butanol (99.5%, RP Normapur, Merck, Darmstadt, Germany), sodium hydroxide (Scharlau, Sentmenat, Spain), sodium acetate (Scharlau), pepsin (Merck), dialysis bags (8–14 kDa molecular weight cutoff), molecular weight markers (Bio Rad Precision Plus Protein Kaleidoscope Prestained Protein Standards and Dual Xtra Standards), Protein Gel Criterion Tris-HCl Precast Gels (Bio Rad, Hercules, CA, USA), Laemmli sample buffer (Bio Rad), tris/glycine/SDS buffer (Bio Rad), 2-mercaptoethanol (Bio Rad), sodium acetate trihydrate (Merck), citric acid (Merck), chloramine-T (Sigma Aldrich), n-propanol (Unilab, Mandaluyong, Philippines), p-dimethylaminobenzaldehyde (Merck), 70% perchloric acid (AnalaR), and L-hydroxyproline (Merck).

### 2.1. Pretreatment

**Figure 1:**
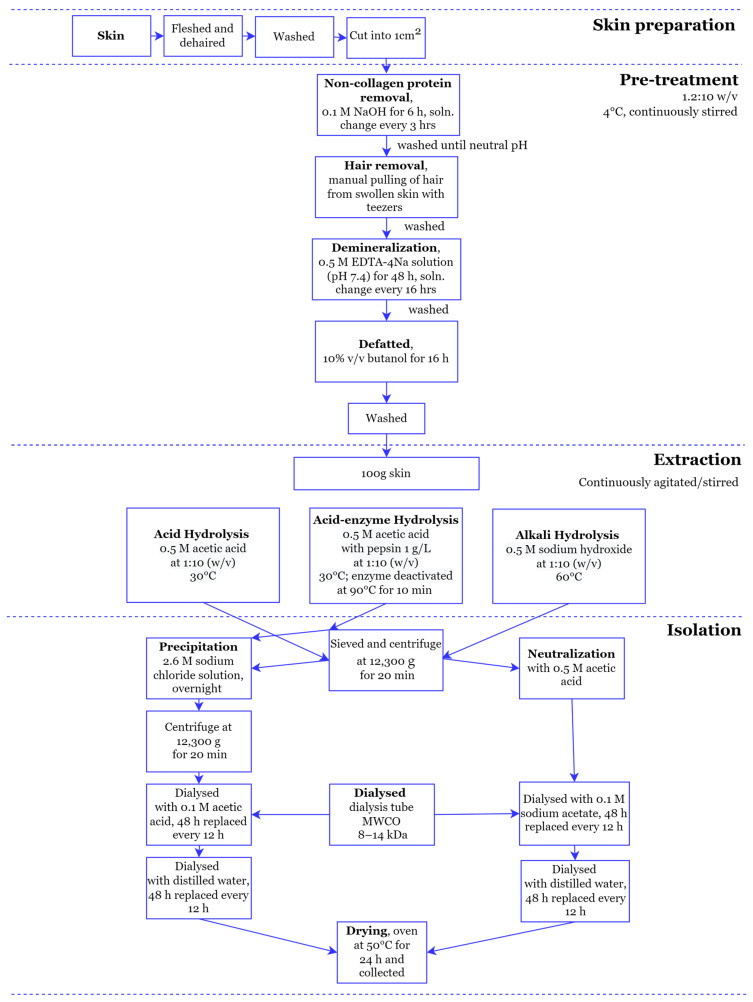
Flowchart of the three extraction methods.

The sheepskins were thawed overnight in a fridge (4 °C). The skins were checked for adhering flesh, fat, and wool and, if present, these were trimmed. The skins were washed and cut into 120 ± 5 g (fresh weight) samples. The pretreatment was conducted at 4 °C with continuous stirring. The various pretreatment steps are shown in Figure 1. All skin samples underwent the same pretreatment with a skin to solution ratio of 1.2:10 kg skin:L solution.

Non-collagenous protein was removed by soaking the skins in 0.1 M NaOH for 6 h; the NaOH solution was changed every 3 h. The skins were then rinsed until the pH of the wash was neutral. The swollen skins were then manually dehaired with tweezers and washed with water. The washed skin was cut into 1 cm^2^ pieces and demineralized by soaking in EDTA-4Na solution (pH 7.4) for 48 h, changing the solution every 16 h. The demineralized skins were washed and defatted by soaking in 10% *v*/*v* butanol for 16 h. The pretreated skin pieces were processed using one of the three extraction methods identified below.

### 2.2. Acid Extraction

The pretreated skin samples underwent acid hydrolysis to extract the acid-soluble collagen. The skins were immersed in 0.5 M acetic acid (a skin-to-acid ratio of 1.2:10 *w*/*v*) at 30 °C for 4–7 days in sealed Erlenmeyer flasks held on a shaker platform (150 rpm agitation speed).

### 2.3. Acid-Enzyme Extraction

For the acid-enzyme extraction, the skin pieces (Section 2.1) were suspended in 0.5 M acetic acid and 1 g L^−1^ pepsin, with a skin-to-solution ratio of 1.2:10 *w*/*v*. This suspension was agitated at 150 rpm at 30 °C for 4–7 days until the skins dissolved then heated at 90 °C for 10 min to deactivate the enzyme.

### 2.4. Alkali Extraction

Alkali soluble gelatin and collagen was extracted by suspending the skins in 0.5 M NaOH with a skin-to-alkali ratio of 1.2:10 *w*/*v*. The suspension was maintained at 60 °C (hotplate), with continuous stirring (150 rpm) for 10–12 h until the skins dissolved.

### 2.5. Isolation of Collagen and Gelatin

The collagen and gelatin were isolated from the extracts of the acid (Figure 1) first by centrifugation (12,300× *g*, 20 min), with the supernatant subjected to salt precipitation by adding sodium chloride to attain a final concentration of 2.6 M, and allowed to stand overnight at room temperature (~23 °C). The precipitate formed was recovered by centrifugation (12,300× *g*, 20 min). The recovered precipitate was dissolved in 0.5 M acetic acid at a ratio of 1:9 *v*/*v*, transferred to a dialysis tube, and dialyzed against 0.1 M acetic acid for 48 h at room temperature. The 0.1 M acetic acid dialysate was replaced with distilled water and dialyzed for 48 h. Both dialysate solutions were replaced every 12 h.

For the alkali extraction, the sample was sieved and centrifuged (12,300× *g*, 20 min) (Figure 1). The supernatant was collected and neutralized using 0.5 M acetic acid. The neutralized sample is dialyzed with 0.1 M sodium acetate (48 h), and distilled water (48 h), with the dialysate solution replaced every 12 h. Sodium acetate rather than acetic acid was used in order to have a similar pH on either side of the dialysis membrane.

The concentrated solution from inside the dialysis bags was placed in trays and dried at 50 °C for 24 h.

### 2.6. Extractable Yield

The extractable yield was calculated as the mass of the dry solids (*M_recovered solids_*) recovered from the extracts divided by the mass of the dry skin (*M_skin_*) used in extraction [12], with weighing taking place immediately after drying.

### 2.7. Fourier Transform Infrared Spectroscopy (FTIR)

FTIR spectra of the dried and ground collagen samples were recorded (Thermo Scientific Nicolet iS5 with iD7 ATR spectrometer; Thermo Fisher Scientific. Waltham, MA, USA) over the wavenumber range of 400 to 4000 cm^−1^ with a resolution of 2 cm^−1^ and data spacing of 0.241 cm^−1^.

### 2.8. Sodium Dodecyl Sulfate Polyacrylamide Gel Electrophoresis (SDS PAGE)

SDS PAGE followed Laemmli’s methodology [13] to determine the molecular weights of the collagen and gelatin extracted by the different methods. The collagen or gelatin was dissolved in the buffer to obtain a concentration of 1 mg mL^−1^. Afterwards, 10 µL of the denatured sample and 5 µL of a protein standard (molecular weight markers) were loaded into wells at the top of a precast gel (4–20% Criterio Tris-HCl Protein Gel, 18 well, 30 µL). Coomassie Blue R250 stain was used.

### 2.9. Hydroxyproline Quantification

Due to the consistent hydroxyproline–collagen ratios in sheepskin, hydroxyproline quantification can be used to provide an assessment of the total collagen and gelatin content [14]. The hydroxyproline in collagen samples was quantified using an adaptation of a published method [15]. Briefly, 0.1 g of a sample (either the extracted collagen or raw skin) in a 50 mL capped vial was digested with 10 mL of 6 M HCl for 24 h in an oven at 110 °C. After hydrolysis, the samples were cooled to room temperature, and 6 M NaOH was added until the pH was 6.0. The resulting solution was filtered, and the volume was adjusted to 50 mL with deionized water. A 50 μL portion of this solution was mixed with 450 μL of chloramine-T (0.056 M chloramine-T in acetate-citrate buffer, pH 6.5) and allowed to stand for 25 min at room temperature in a capped vial. A 500 μL portion of Ehlrich’s reagent was added to the vial and it was held in a 65 °C water bath for 20 min to allow the color to develop. The absorbance value of the sample was measured against a blank (distilled water) in a UV spectrophotometer at 550 nm. The readings were converted to concentration of hydroxyproline using a calibration plot that had been prepared using standard solutions of hydroxyproline.

### 2.10. Small-Angle X-ray Scattering (SAXS)

SAXS diffraction patterns were recorded on the Australian Synchrotron SAXS/WAXS beamline using a high intensity undulator source. The energy resolution was 10^−4^ using a cryo-cooled Si(111) double crystal monochromator. The beam size (FWHM focused on the sample) was 250 × 80 µm with a total flux of approximately 2 × 10^12^ photons s^−1^. A Pilatus 1 M detector with an active area of 170 × 170 mm, a sample detector distance of 3371 mm, and an X-ray of 8 or 11 keV, was used to record all the diffraction patterns. The exposure time for diffraction patterns was in the range of 1–5 s. Data were processed using Scatterbrain 2.82 software [16].

## 3. Results and Discussion

### 3.1. Extracted Collagen

**Figure 2:**
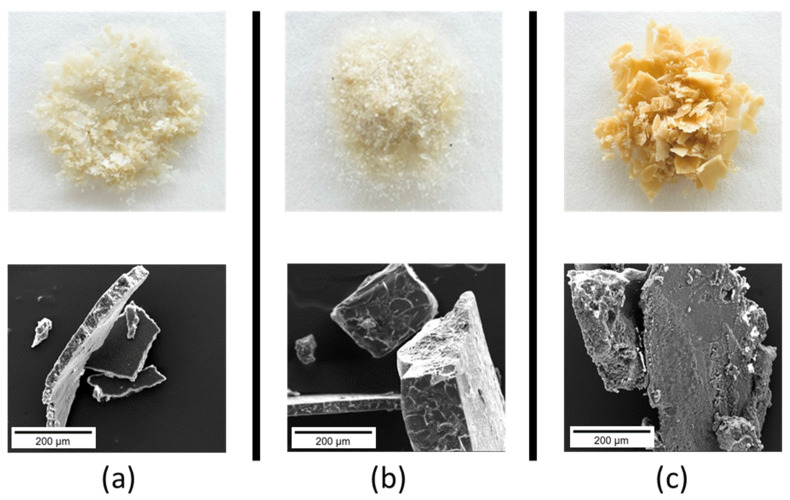
Dried gelatin–collagen (top, optical images; bottom, SEM images) from the different extraction methods: (**a**) acid hydrolysis, (**b**) acid-enzyme, (**c**) alkali hydrolysis.

The samples extracted using the different methods differed in appearance and texture (Figure 2). The acid-extracted samples had a lighter off-white appearance (Figure 2a,b) compared to the alkali-extracted sample (Figure 2c). The acid processed samples comprised larger aggregates with smoother surfaces and sharp edges, typical of gelatin (Figure 2, SEM images), whereas the alkali processed sample had solid particles with rougher surfaces.

### 3.2. Extractable Yield

The extraction yield of gelatin–collagen using the different methods are as follows (average values ± standard deviation (% *w*/*w*); three replicates): 3.1 ± 1.6 for acid extraction; 4.8 ± 1.9 for acid-enzyme extraction; and 4.1 ± 7.7 for alkali extraction (Table 1). Based on the Student’s t-test, the yields from the acid and acid-enzyme extractions were not significantly different (*p* > 0.05). Including enzymes had no positive benefit on the extraction yield, which is in contrast to other studies that have shown improved extraction of collagen from cattle skin and fish skin in the presence of enzymes [12,17].

### 3.3. Hydroxyproline Quantification

Collagen molecules have a specific characteristic motif of Gly-X-Y (Gly = glycine, X = proline, Y = 4-hydroxyproline) that stabilizes their triple-helix structure. The hydroxyproline component in the motif is also uniquely abundant in collagen compared to other proteins. The abundance of hydroxyproline in an extract is used when estimating its collagen content. The measured data (Section 2.10) were used in the following equation to calculate the hydroxyproline content:

(1) $$Hydroxyproline\;(\mu g\;g^{-1}\;sample)=\frac{Hydroxyproline\left(\mu g\;mL^{-1}\right)\times Dilution\;volume\;(mL)}{Sample\;weight\;(g)}$$

The collagen content was then estimated using the following equation:

(2) $$Collagen\;content\;\left(\mu g\;g^{-1}\;sample\right)=\frac{Hydroxyproline\left(\mu g\;g^{-1}\;sample\right)\times Conversion\;factor}{1000}$$

For sheepskin collagen extracts and the raw sheepskin samples, it is known that ca. 12.5% of the collagen mass is hydroxyproline [18]. This is slightly less than in the hides of other animals such as cattle, deer, and goat. Using the percentage of hydroxyproline in sheepskin, the collagen content of the samples was calculated using a conversion factor of 8 (100/12.5) in Equation (2). The hydroxyproline and collagen content of the samples are shown in Table 1. The material extracted by acid extracted material contained the most gelatin–collagen. The acid extract had 7.6-fold more gelatin–collagen than the gelatin–collagen content of the alkali-processed sample (Table 1). Although the alkaline solution had a high extraction of material, it is also known that the alkali treatment will readily hydrolyze collagen into smaller peptides [19]. The relatively high extraction temperature (60 °C) for the alkali method may also have been a factor in hydrolyzing the collagen into small molecular weight components. These small molecular weight components would have been mostly removed during dialysis using the membrane with a molecular weight cutoff of 8–14 kDa; and therefore, are not included in the apparent yield. Nevertheless, such small molecular weight components are not useful for most gelatin applications; and therefore, it is useful not to include this component in the apparent yield calculation.

The hydroxyproline contents in the materials produced by acid and acid-enzyme extraction here are similar to or greater than those which have been reported for cattle skin [12,20] and fish skin [21] subjected to similar treatments.

The extraction yield from sheepskin is marginally lower than that obtained from cattle hide [12]. Notably, sheepskin has a higher fat content (30–50% in the dermis alone) compared to bovine hide (2–3% fat) and goatskin (3–10% fat) [22]. The extra fat, which was not effectively removed in the defatting stage, may have contributed to the lower extraction yield of collagen by providing the collagen with some protection (by hydrophobicity) from the digestion chemicals.

The extraction yield of gelatin–collagen from skin could perhaps be improved by modifying the pretreatment conditions to better “open up” the skin matrix, enabling better penetration of the hydrolysis chemicals. In the leather industry, an initial stage of liming the skins and hides is performed to remove the hair, solubilize the fat, and swell the skin, which opens up the fibers for better chemical penetration at subsequent processing stages [23].

### 3.4. FTIR

**Figure 3:**
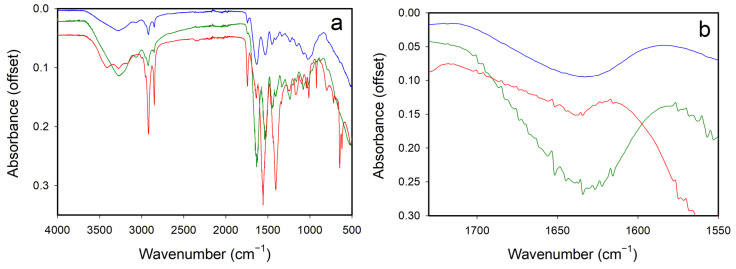
FTIR spectra of collagen recovered by acid (blue line), acid-enzyme (green line), and alkali (red line) extraction: (**a**) full spectrum; (**b**) expanded region that assists in distinguishing between gelatin and collagen [24]. See Table 2 for details of peak assignments.

The FTIR spectra of the material extracted using the different extraction protocols had some major differences (Figure 3). A summary of the positions of the main absorption bands and their assignment is provided in Table 2. Absorption bands that can be assigned to collagen or gelatin and bands due to keratin are observed in all samples, but the proportions of each differs between the samples. The material from the alkali extraction contained more intense peaks, which is due to the keratin.

Whether the material contains collagen or gelatin can be observed from the amide I bands, which are from C=O stretching in hydrogen-bonded carbonyls of proteins. These bands are sensitive to changes in the secondary structure of proteins. Amide II bands from C–N stretching are, to a lesser extent, also affected by changes in the secondary structure of proteins [27]. Amide III bands result from C–N and N–H planar deformation vibrations and other effects associated with carbon-nitrogen stretching and the bending of the nitrogen-hydrogen. These bands are sensitive to changes in the triple helix structure and the polarity of the protein.

To distinguish between collagen and the hydrolysis product gelatin it has been shown elsewhere that the amide I absorption bands between 1700 and 1600 cm^−1^ can be used as described in the literature [24]. Collagen has been shown to have a peak at 1659–1651 cm^−1^, whereas in gelatin the peak is at 1643–1633 cm^−1^, depending on the temperature during the measurement (lower wavenumber at higher temperature). This region of the FTIR recorded here is displayed in Figure 3b. In these samples there is just one broad peak centered at 1630 cm^−1^ for the acid and acid-enzyme extracted material and a peak at 1638 cm^−1^ for the alkali extracted. These may indicate that gelatin dominates over collagen in all of these extracted materials and, due of the shift in band position, could suggest that the alkali extracted material is hydrolyzed further than the acid extracted material, although the poor resolution of this region limits the conclusions that can be drawn.

The FTIR makes it clear that these materials are not pure collagen or pure hydrolysis products of collagen. There are intense peaks associated with keratin, and these are more prominent in the alkali extracted material. The low hydroxyproline content of the alkali material showing that it is less than 8% gelatin and the intense FTIR peaks for keratin show that the alkaline extracted material is likely to be largely keratin.

### 3.5. SDS PAGE

**Figure 4:**
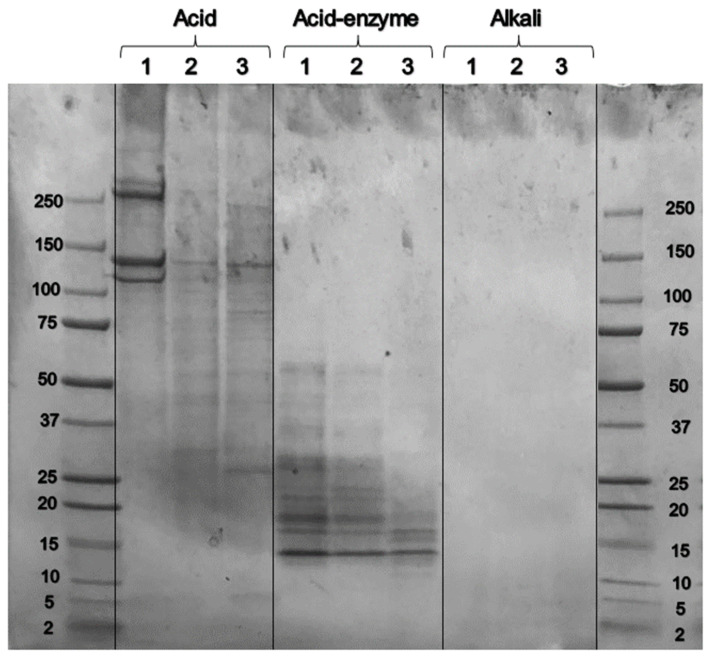
SDS PAGE of collagen from acid, acid-enzyme, and the alkali extraction processes. The lanes on extreme left and right show the globular protein molecular weight markers (2–250 kDa). There are three replicate lanes for each treatment. The figure has been modified to remove some lanes on the right-hand side not discussed in this work (full original SDS PAGE available).

The developed SDS PAGE gel shows the molecular weights of the extracted proteins (Figure 4). The globular protein molecular weight standards are known to be unfavorable molecular weight standards for the fibrous collagen components, possible due to different SDS binding, so are presented only as an approximate range standard. Collagen extraction by acid hydrolysis resulted in products with molecular weights in the range of 100 to greater than 250 kDa. When enzyme was used in conjunction with the acid hydrolysis, much smaller molecular weight components resulted, mostly 13–30 kDa. For the alkali extraction process indistinct bands were observed in the SDS PAGE.

The simple acid hydrolysis therefore results in material that contains more of the longer collagen chains and less material that has been broken into smaller fragments of gelatin. The α1-chains and α2-chains, specific to collagen-1, produce bands at around 116 kDa [11], as observed here. Furthermore, the higher molecular weight bands observed here are the result of either two crosslinked α-chains (β-band), or three crosslinked α-chains (γ-band).

The acid hydrolysis with enzymes results in the collagen being hydrolyzed into small fragments, in other words, gelatin. The dialysis membrane used for separation after extraction had a cutoff of 8–15 kDa so the range observed here in the SDS PAGE of 13–30 kDa is limited by this separation and it is probable that smaller polypeptide fragments were also produced by the acid-enzyme hydrolysis. Although pepsin is considered to digest only the non-helical parts of the procollagen [28], acid hydrolysis (as used here) is nonspecific.

The samples extracted with alkali, did not have distinct bands in the SDS PAGE. However, from the hydroxyproline analysis and the FTIR, it was clear that this material was largely keratin and contained only a small proportion (7.8%) of collagen or collagen hydrolysis products. In other studies, alkali-based treatments have been found effective for extracting collagen under milder conditions than in the present work [29].

Current market conditions suggest that “collagen peptides” or “collagen hydrolysate” may represent an increasing market sector wherein the smaller components have distinct value. If these components could be efficiently recovered by methods such as those studied here, high-quality peptides may be produced in greater quantities and higher value than low bloom strength gelatin.

### 3.6. SAXS

**Figure 5:**
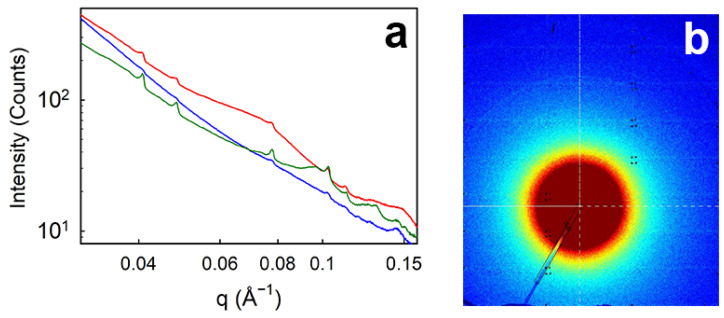
(**a**) SAXS integrated scattering intensity for the extract obtained by acid (blue line), acid-enzyme (green line), and alkali (red line) extraction, showing sharp peaks for diffraction from the collagen fibril D-banding; (**b**) an example of the SAXS pattern (acid extracted material) with the faint rings visible due to the collagen diffraction (the color scale represents diffraction intensity).

The SAXS diffraction patterns of the gelatin–collagen material recovered by the three extraction methods are shown in Figure 5. The diffraction peaks (Bragg peaks) are due to banding in the fibrillar structure of collagen [30]. The presence of this diffraction feature is definitive evidence for the presence of collagen fibrils in the samples. The distance between the bands (i.e., the *D*-spacing) in the collagen molecules is calculated from the diffraction data. Diffraction also occurs from the triple-helix, at around 0.287 nm [31]. Triple-helix structures exist in gelatin or collagen-like peptide fragments [32]. We did not measure diffraction at this scale, only the diffraction angle at which we can see fibrillar banding at around 63 nm.

The presence of these D-spacing diffraction peaks in the acid-enzyme and the alkaline samples is a little surprising considering the SDS PAGE results showing little material of higher molecular weight in these samples (although there is high molecular weight material in the acid extracted material). This perhaps is an indication of the sensitivity and specificity of SAXS for detecting fibrillar collagen.

The *D*-spacing is barely affected by the method of collagen extraction (Table 3) and is similar to that present in native tissues of various types, for example [33].

### 3.7. Commercialization

The adaptation of this process for commercialization would require some changes to what is described here in this laboratory study. For example, demineralization with EDTA is not required and the initial processing of the skins could be a conventional beamhouse operation in which the skins are dehaired and defatted to produce pickled pelts.

## 4. Conclusions

This study trialed three different methods for extracting collagen from sheepskin. The key difference with sheepskin from other skins is the high fat content that must be removed at the start of the process. A gelatin–collagen mix was successfully extracted using both an acid process and an acid-enzyme process. All three approaches give distinct and different products with the potential to adapt and give three different commercially relevant materials. Longer chain polypeptides were obtained from the acid process, while the acid-enzyme process broke the collagen into small fragments considered to be gelatin. An amount of fibrillar collagen was observed in all extracts. The alkaline extraction was dominated by keratin which may find markets in haircare. Small angle X-ray scattering, was shown to be sensitive in detecting small quantities of intact fibrillar collagen in the extracts. The range of collagen derived molecules with varied molecular weights in these extracts, and the ability to select this range, informs of the uses of these materials in varied applications.

## Figures and Tables

**Table 1:** Hydroxyproline and gelatin–collagen content of the material recovered using the different extraction methods (acid, acid-enzyme, alkali) and in raw sheepskin.

Sample	Hydroxyproline Content (mg g^−1^ Dry Product)	Gelatin–Collagen in Dry Product (%)	Gelatin–Collagen Extracted (mg g^−1^ Dry Raw Skin)	Extracted Gelatin–Collagen Relative to the Total Amount of Collagen in the Raw Skin (%)
Acid	74.2 ± 8.1	59.3 ± 6.5	10.7	3.1
Acid-enzyme	85.1 ± 25.3	68.1 ± 20.2	16.8	4.8
Alkali	9.7 ± 2.1	7.8 ± 1.7	14.3	4.1
Raw sheepskin	19.4 ± 7.3	15.5 ± 5.8	-	-

**Table 2:** FTIR absorption bands of amide vibration modes from the literature [25] and measured in this work, and the keratin bands identified in these samples which were assigned using the literature [26].

Band	Literature Band (Wavenumber cm^−1^)	Absorption Band Positions from This Work for Different Extraction Methods (Wavenumber, cm^−1^)			Functional Group and Vibration Modes
		Acid	Acid Enzyme	Alkali	
Collagen and gelatin					
Amide A	3300–3500	-	-	-	NH stretching
Amide B	~3100	3065	3065	3163	NH stretching
Amide I	1600–1800	1630	1635	1638	C=O stretching
Amide II	1470–1570	1530	1528		CN stretching
Amide III	1250–1350	1334	1334	1339	CN stretching, NH bending
Keratin specific (keratin also displays amide bands above)					
	2931	2921	2923	2919	(CH_3_) symmetric stretch
	2875	2851	2852	2850	(CH_2_) symmetric stretch
		1743	1743	1743	unassigned
	1551			1559 (overlaps with collagen band)	(NH) bend; (CN) stretch
	1420	1409	1407	1408	(CH_3_) deformation
Water					
		3275	3275	3275	OH stretching (water)

**Table 3:** The *D*-spacing of collagen extracted by acid or alkali.

Extraction Method	*D*-Spacing (nm)
Acid	63.0
Acid-enzyme	63.0
Alkali	62.9

## Data Availability

Data are contained within the article.
